# Robotic Hepatectomy plus Biliary Reconstruction for Bismuth Type III and Type IV Hilar Cholangiocarcinoma: State of the Art and Literature Review

**DOI:** 10.3390/jpm14010012

**Published:** 2023-12-21

**Authors:** Simone Guadagni, Annalisa Comandatore, Niccolò Furbetta, Gregorio Di Franco, Cristina Carpenito, Bianca Bechini, Filippo Vagelli, Niccolò Ramacciotti, Matteo Palmeri, Giulio Di Candio, Luca Morelli

**Affiliations:** 1 General Surgery Unit, Department of Translational Research and New Technologies in Medicine and Surgery, University of Pisa, 56126 Pisa, Italy; simone5c@virgilio.it (S.G.); a.comandatore@libero.it (A.C.); gregorio.difranco@med.unipi.it (G.D.F.); cristina.carpenito@gmail.com (C.C.); bianca.bechini@gmail.com (B.B.); filippovagelli93@gmail.com (F.V.); n.ramacciotti94@gmail.com (N.R.); palmeri.matteo@gmail.com (M.P.); giulio.dicandio@unipi.it (G.D.C.); luca.morelli@unipi.it (L.M.); 2 EndoCAS (Center for Computer Assisted Surgery), University of Pisa, 56126 Pisa, Italy

**Keywords:** Hilar Cholangiocarcinoma, da Vinci, robotic liver resection

## Abstract

Background: In Bismuth type III and IV Hilar Cholangiocarcinoma (III–IV HC), surgical resection is the only chance for long-term survival. As the surgical procedure is complex and Robotic-Assisted Surgery (RAS) may be particularly suitable in this setting, the aim of this study is to evaluate the potential benefits of RAS in III–IV HC in terms of post-operative outcomes. Methods: We conducted a systematic review using the PRISMA checklist for article selection. We searched the PubMed database and included only studies with clinical data about the treatment of III–IV HC using RAS. Results: A total of 12 papers involving 50 patients were included. All cases were Bismuth IIIa (*n* = 18), IIIb (*n* = 27) or IV type (*n* = 5) and underwent hepatectomy with biliary confluence resection and reconstruction. The mean operative time was 500 minutes with a conversion rate of 4%. The mean hospital stay was 12.2 days, and the morbidity and 30-day mortality rate were 61.9% and 2%, respectively. Over a mean follow up period of 10.1 months, 9/18 cases experienced recurrence (50%). Conclusions: RAS for III–IV HC is safe and feasible, at least if performed by experienced surgeons on selected cases. The oncological outcomes appear acceptable, given the aggressiveness of this pathology, but further studies are needed to fully elucidate the exact role of robotics in this setting.

## 1. Introduction

Hilar Cholangiocarcinoma (HC) is an uncommon hepatobiliary tumor that arises between the second-order bile ducts and the insertion of the cystic duct into the common bile duct. Due to its aggressive behavior and the absence of early symptoms, most individuals are diagnosed with locally advanced or metastatic disease. Multidisciplinary evaluation is mandatory as a sub-group of patients may be candidates for liver transplantation, while others are directed to receive only chemotherapy, which has limited efficacy [1].

On the other hand, approximately 25% of all cases may be eligible for surgical resection, offering the potential for long-term survival with R0 margins. Particularly for type III and type IV (III–IV HC), according to Bismuth classification, surgery is highly challenging, even with an open approach, and has been associated with a mortality rate reported in several studies up to 18%, while morbidity ranges from 27 to 54% [2,3,4].

In the last ten years, the Robotic-Assisted Surgery (RAS) has expanded the feasibility of minimally invasive surgical procedures and has shown improved outcomes in several surgical areas. For example, it has been shown to be effective in rectal resection [5] and in pancreatic resection [6], as demonstrated in various studies. The resection of III–IV HC can serve as a valuable testing ground for RAS due to the combined challenges of achieving clear surgical margins, controlling hemorrhages, finely isolating of hilar structures and dealing with complex anastomosis. From a technical perspective, this type of surgery could benefit from RAS, as accumulating evidence has demonstrated its non-inferiority compared to laparoscopy even in terms of oncologic outcomes [7].

The objective of the present study is to perform a systematic review of peri-operative and oncologic results of RAS in III–IV HC and to summarize the current evidence regarding safety and feasibility of this complex surgery.

## 2. Materials and Methods

We followed the guidelines outlined in the Preferred Reporting Items for Systematic Reviews and Meta-Analyses (PRISMA) statement for conducting and reporting our selected articles [8]. Two authors (SG and LM) independently carried out all the steps, searching the PubMed and Scopus database for articles published between January 2009 and August 2023. The search strategy involved various combinations of keywords such as “hilar cholangiocarcinoma OR perihilar cholangiocarcinoma OR Klatskin tumor” AND “da Vinci OR robotic OR robotic-assisted surgery” AND “liver resection”. Only articles written in English were considered. Additionally, we conducted the extensive cross-checking of the reference lists of all retrieved articles that met the inclusion criteria to further expand our search.

### 2.1. Study Selection

We initially screened the abstracts as well as titles of all the manuscripts, followed by a comprehensive evaluation of the full text. The target population consisted of patients with III–IV HC who underwent robotic hepatectomy and biliary reconstruction. In case series where various type of HC were discussed, we only extracted data about the type III–IV sub-group. We included left hepatectomies, right hepatectomies and segments V–IV resection associated with segment I resection and primary or secondary order bile duct anastomosis. We applied the PICOT (Population, Intervention, Comparison, Outcome and Time) framework to define criteria for selecting articles. The following exclusion criteria were applied:

1. Original studies that did not report perioperative and oncologic outcomes for these patients.
2. Review articles, letters and comments.
3. Studies from which it was impossible to retrieve or calculate the relevant data.

### 2.2. Data Extraction

The two reviewers (SG and LM) collected and extracted the following data: first author, year of publication, study type, number of patients involved, lesion’s classification according to Bismuth sub-types, age, sex (male/female), body mass index (BMI), neoadjuvant therapy, pre-operative biliary drain, portal vein ligation (PVL), embolization (PVE) or Associating Liver Partition and portal vein ligation for staged hepatectomy (ALPSS), type of operation, total robotic trocars used, number of bilio-jejunostomy ducts, operative time, blood loss, length of hospital stay (LOS), conversion rate, overall morbidity, morbidity > 3 according to the Clavien–Dindo classification [9], re-operation rate, biliary leak according to the International Study group of Liver surgery [10], TNM staging, resection margins, number of harvested lymph nodes, 30-day postoperative mortality, mean follow-up, recurrence rate and deaths during follow-up.

### 2.3. Quality Assessment and Statistical Analysis

We assessed the methodological quality of the studies using various tools, including the Oxford Centre for Evidence Medicine’s critical appraisal tool, checklists of the Dutch Cochrane Centre, BMJ editor’s checklists and the checklists of the EPPI Centre [11]. The studies were rated according to their overall quality as very low, low, moderate or high. Due to the heterogeneities in reporting certain outcomes across the included studies, global data pooling was not feasible. However, we calculated means for case reports and case series that reported outcomes for each single patient. Statistical analyses were conducted using Excel software (Microsoft corporation, Office version 2021).

## 3. Results

**Figure 1:**
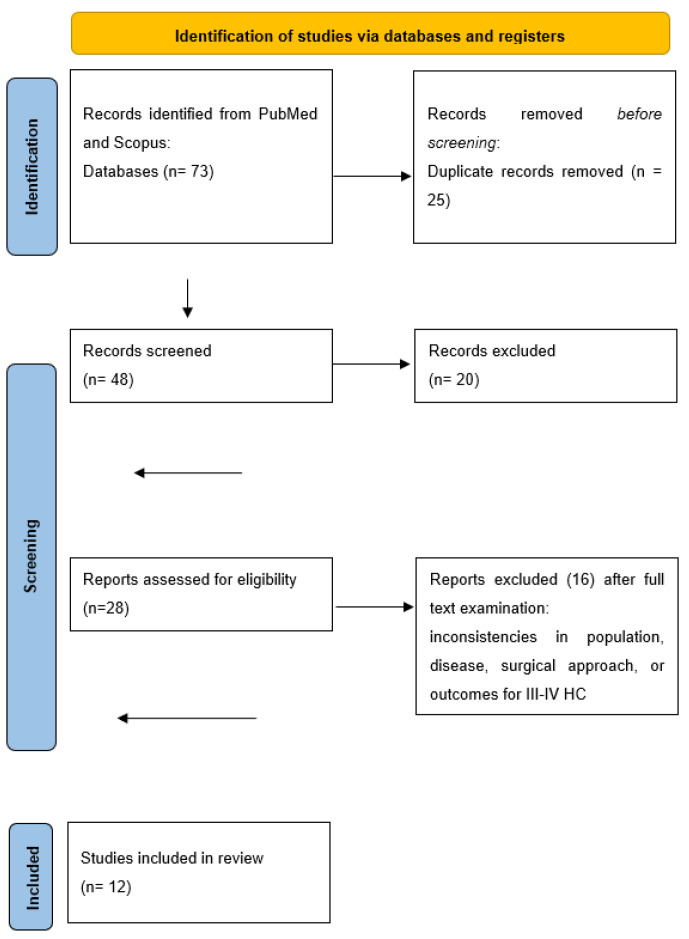
PRISMA flow diagram for our systematic reviews.

The initial literature research yielded 73 English papers. After removing duplicates, and screening titles and abstract, we assessed 28 studies through full-text evaluation. Sixteen manuscripts were subsequently excluded for the various reasons including inconsistencies in the population, disease, surgical approach or outcomes for III–IV HC. Ultimately, we included a total of 12 papers [12,13,14,15,16,17,18,19,20,21,22,23], reporting the outcomes of 50 patients, in this review (Figure 1). Nine of these papers are case reports, while three are retrospective single-center experience, with the largest series consisting of 28 patients. In this largest series, 48 cases were described, but we excluded 20 cases that were Bismuth types I and II and underwent only biliary resection. No randomized trials were found. All the results are summarized in Table 1, Table 2 and Table 3. Regarding the level and quality assessment of each included study, according to the critical appraisal tool of the Center for Evidence-Based Management—CENMa, all of them were considered to be level IV and of low (3/12; 25%) or very low quality (9/12; 75%).

We extracted age and gender information from all articles, while BMI, neoadjuvant therapy and pre-operative biliary drainage were present in three, eight and seven articles, respectively. The mean age across all case reports and the two-case series [14,19] was 59.9 years, while in the study by Li et al. [18], the mean age for the entire cohort was 63 years. According to the Bismuth classification, eighteen cases were classified as type IIIa, twenty-seven as type IIIb and only five as type IV. In four articles, we found information about portal vein embolization or ALPSS to prevent post-hepatectomy liver failure.

The da Vinci version used was reported in 9/12 articles (75% of total). The da Vinci Si version was utilized in four articles, while the newest Xi version was used in the three most recent articles. Only one article performed the operation using the S version, whereas only one other used the da Vinci X versions. All authors, except one, used four robotic trocars. The type of surgical intervention depended on the pattern of intra-ductal tumor’ growth and included left or right hepatectomy or bi-segmentectomy (Taj Mahal procedure, described only in one article), plus segment I resection and hilar lymphadenectomy. The reconstructive phase involved Roux-en-Y bilio-enteric anastomosis, with only four authors [19,20,21,22,23] reporting more than one bile duct anastomosis (mean 2.12 ductal anastomosis). The operative time was extracted in 92% of cases, and considering all case reports and two case series, the mean operative time was 500 minutes. In the remaining case series [18], we were unable to extrapolate the operative time for only the III–IV HC patients, as the authors reported a median of 276 minutes of console time for the entire cohort. Blood loss was reported in all manuscripts except in one, with a mean of 623 mL. Similar to operative time, we could not extrapolate the blood loss of the III–IV HC patients in Li et al. [18] study, which stated a median of 150 mL. Conversion to traditional surgery was required in one case report [15] and one patient in a case series [19], resulting in a conversion rate of 4%. The reason of conversion was defined only in the case series and was related to the short mesentery preventing the jejunal loop from being pulled up enough for a tension-free anastomosis.

Only one study did not provide information regarding hospital stay. The mean hospital stay was 12.2 days, excluding the report by Li et al. [18], which stated a median hospitalization of 9 days (range 4–52 days) for the entire series that included also type I and type II, and we were not able to extrapolate data only for type III HC. Data on morbidity and 30 days-mortality were extracted from all works except one; the morbidity rate was 13/21 (61.9% of total), whereas Li et al. [18] reported a morbidity rate of 28/48 (58.3%) for the whole series. The Clavien–Dindo grade ≥3 was 3/21 (14.2%), whereas Li et al. [18] reported a 5/48 (10.4%) for the whole series. Re-operation was described only by Xu et al. in two patients: one who required portal trombectomy and anastomosis and another who underwent arterial reconstruction after the failure of interventional radiology due to a pseudoaneurysm. A biliary leak was described in 4/21 (19%), while Li et al. [18] reported a 2/48 (4.2%) biliary leak rate for the entire series. All cases were treated conservatively, except one case that was grade B and required percutaneous imaging guided drainage. Mortality rate was 1/50 (2%) due to liver failure [14].

From a pathological standpoint, the mean number of harvested lymph nodes was 12.2 described in only five articles [15,17,19,21,22]. Similarly, TNM staging was reported in six articles as follows: T1N0 (one case), T2N0 (two cases), T2N1 (one case), T3Nx (one case), T3N2 (one case), T4N0 (one case) and T4N1 (two cases). R0 margins were achieved in 17/22 (77.2%) and Li et al. [18] reported a R0 rate of 73% for the whole series. The mean follow up was 10.1 months (range 5–21.4 months) retrieved in seven articles [12,14,16,19,20,21,22], with a recurrence rate of 9/18 (50%). Insufficient data and the heterogeneity of the study sample or metrics employed did not permit a cumulative analysis of disease and overall survival.

## 4. Discussion

HC is a tumor characterized by an aggressive behavior and consequently with a low survival rate [24]. Even among patients with radiological localized disease, a smaller number of cases are suitable to R0 radical resection due to a high incidence of local invasion as well as involvement of major hepatic vessels, as well as the microscopic intra-ductal grow. Thus, very aggressive surgical treatment is generally required to increase the chances of long-term survival. Particularly for type III or type IV subgroups, detailed pre-operative planning is mandatory with liver remnant measures, because the surgical procedure, as well as the resection of the biliary confluence, consists mostly of an en-bloc major hepatectomy, with a consequent risk of post-operative hepatic disfunction [25]. The other crucial phase in this situation is the hilar one with a clear definition of all biliary ductal branches and the involved vessels, which can also be difficult to outline during open surgery.

Compared to other liver malignancies, the minimally invasive treatment of HC, especially type III and IV, is a relatively novel field. Laparoscopic resection is still not universally considered as an acceptable option respect to the standard open procedure, primarily because of the challenges faced during major hepatic resection, the lack of consensus regarding the oncological adequacy of this approach, and the difficulties in the reconstruction phase due to the kinematic limitations of the technique [26]. The development of robotics in general surgery has made several operations simpler in a minimally invasive fashion, so for the treatment of III–IV HC it has become an appealing alternative.

As a first step, an increasing number of studies have shown several advantages of robots with respect to laparoscopy in the field of hepatic surgery. For instance, a recent multicentric propensity score analysis [27] that compared RAS versus laparoscopy in hepatic resection showed a reduced conversion rate and low blood loss, especially in difficult cases. Subsequently, some authors also started treating HC with or without associated hepatic resection and reconstruction with RAS [28].

The present review focuses on 50 III–IV HC patients treated with RAS in the 14-year analyzed period.

The first report by Giulianotti et al. [12] in 2010 demonstrated the feasibility of robotic right-side hepatectomy, biliary confluence resection and left-side bilio-enteric anastomosis in a type IIIa HC. The authors emphasized the superiority of RAS compared to standard laparoscopy in this setting, with particular attention to the reconstructive phase in which the Endowrist technology and filter tremor make a difference during the delicate and small biliary ductal isolation and anastomosis. Moreover, they also highlighted how precise dissection and magnified vision increase the control of small arterial and portal branches, both at the hilar plate and during the hepatic resection. After this first case, the following early experiences using the da Vinci S or Si versions were still isolated case reports, with the exception of Li et al. [18], whose study concerned in a high volume hepato-bilio-pancreatic center.

The introduction of the da Vinci Xi in 2015 further changed the panorama of minimally invasive surgery, as it drastically improved the workflow of operations in a full robotic manner. Indeed, aiming to overcome the limitations of the previous version, the Xi system presented new important features such as an increased flexibility and a new overhead arrangement of the robotic arms, the magnetic connectors and the rotating boom as well as an 8 mm high resolution camera that can “hop” throughout all trocar. Thanks to these characteristics and technologies, docking has become easier and faster and the workspace range has been increased [29]. As a consequence, in the second study period of our review, an increasing number of authors have used the da Vinci Xi version, publishing case series of type III–IV HC treated with RAS [19].

Cillo et al. [19] in 2021, reporting a case series operated using the da Vinci Xi, focused on clear inclusion criteria for RAS in this setting and particularly emphasized the importance of the planned left hepatectomy (type IIIb), the absence of vascular invasion and the absence of previous upper abdominal surgery. In contrast, Li et al. [18], in their series, excluded patients with other malignancies and patients treated with palliative surgery or HC type IV. Considering this last aspect, only two articles in our review dealt with Bismuth type IV HC. Of these, Xu et al. [14] treated four type IV HC patients in their series of nine patients. They performed three left hepatectomies and one right hepatectomy plus a caudal lobe resection and a biliary reconstruction corresponding to the intra-ductal grow predominance of tumor. However, two of these patients (50% of total) had a positive resection margin.

Another different approach described in the present review is the Taj Mahal procedure [30], which consists of I, IV and V segment resection with right/left biliary reconstruction. It differs from conventional operations as it reduces the damage to normal liver tissue, reducing the rate of organ failure while maintaining the oncological efficacy. On the other hand, the Taj Mahal procedure requires two plains to remove these liver segments, as well as the dissection of the hepatic artery and portal vein using an intra-Glissonian approach. This option is seldom reported, but Deng et al. also described the utility of the robot also for Taj Mahal procedure [23], confirming that it offers a further option in selected cases to perform minimally invasive hepaticojejunostomies even when multiple ducts are present.

The operative time reported in the articles selected for the present review is quite variable, but several factors could explain this heterogeneity. Firstly, the case reports included may describe the first experiences in several centers. In addition, the operative time can be influenced by the type of hepatic resection performed and the number of biliary duct anastomosis. The largest series described by Li et al. [18] showed a median console time of 276 min with a blood loss of 150 mL; although the experience included biliary resection without hepatectomy, it demonstrated that after the initial learning curve the operative time becomes closer to open surgery reported in several works [31].

The conversion rate was 4% in total. These data are in line with another recent systematic review on all types of HC treated with minimally invasive surgery [25]. In this latter paper, which however also included easier cases (e.g., type I and II), robotic approaches had a conversion rate of 1.9%, while laparoscopy had a conversion rate of 11.6%. This very low conversion rate in challenging operations performed using da Vinci robot is consistent with literature, as has been already reported in several other setting. This aspect, together with the shorter learning curve [26], seems to be one of the major advantages of robotic assistance as it translates to an expansion minimally invasive techniques.

Similarly, the post-operative course of all patients in this review appears favorable, considering that the overall morbidity and mortality rates are 58–61.9% and 2%, respectively, versus open approaches, which are 14–71% and 0–17% in other large studies [32]. Quite interestingly, biliary leaks are reported only in three works with a rate of 4–19%. This demonstrates that even though the absence of haptic feedback from the robot may be a disadvantage, the experience of the operating surgeon, the magnified vision and the degree of instrument’ freedom mitigate this problem, even in small anastomosis. Furthermore, particularly focusing on the biliary reconstruction, Sucandy et al. [20] described the use of 3-0 barbed sutures in performing their hepatico-jejunostomy. We underline this point, as in several fields of the robotic panorama, the use of self-fixing barbed sutures has become popular, as the suture, once tightened, maintains its tension after every passage. A continuously constant tension is thus assured, despite the lack of retraction, and this is important, especially in delicate tissues such as pancreato or hepatico-jejunostomy [33].

The oncological outcomes are also crucial as they determine mid- and long-term survival. Our analysis reveals a R0 rate of 73–77%, which may be a very good result when compared to HC larger series of open surgery [34,35]. Data on survival and recurrence are limited and mostly based on short follow-up periods. Out of the twelve studies, seven reported on oncological follow-up, resulting in a total mean recurrence rate of 50%, with a maximum of 88% by Xu et al. [14]. These outcomes are in line with studies that evaluate recurrence rate after open surgery for HC [36].

The present study has certain inherent limitations that inevitably affect the final results of the review. The main limitation is related to the small number of studies included, most of which are case reports and therefore have a high risk of bias. Additionally, the overall number of treated patients is very limited, with only three case series (with a maximum of 28 patients), and there is heterogeneity in the experiences of the different operators over the long study period considered. Moreover, the absence of case–control groups and comparative results precludes any possibility of conducting meta-analyses or quantitative evaluations. However, because of the complexity and rarity of the type III and IV hilar cholangiocarcinoma, only few surgeons are simultaneously highly skilled in both hepatobiliary surgery and robotic surgery and have performed robotic hepatectomy and biliary reconstruction for type III and IV hilar cholangiocarcinoma so far. Hence, to describe the state of the art on this topic, it was necessary to include all reported cases. As a result, the present review is the first to summarize the current literature on the robotic treatment of type III–IV HC.

In conclusion, although open surgery remains the most commonly employed, RAS appears promising and capable of expanding minimally invasive procedures in this highly challenging setting. RAS for type III–IV HC is safe and feasible, at least if performed by experienced surgeons on selected cases. The oncological outcomes seem to be acceptable, given the aggressiveness of this pathology, but further studies are needed to fully elucidate the exact role of robotics in this setting.

## Figures and Tables

**Table 1:** Studies on robotic surgery for III–IV HC: study, patients and procedural characteristics.

Author	Year	No. of Patients	M/F	Age (Years)	BMI (kg/m^2^)	Bismuthtype	Neo-Adjthy	Pre-Op BD	Pre-Op PVE/ALPSS
Giulianotti [12]	2010	1	1 M	66	23	3a	0	1	1 PVE
Zhu [13]	2014	1	1 M	43		3a	0	1	1 PVE
Xu [14]	2016	9	7 M 2 F	58 ^#^		IIIa 4 IIIb 1 IV 4	0	1	1 PVE
Quijano [15]	2016	1				IIIb			
Machado [16]	2020	1	1 F	76	30	IIIb	1	1	
Marino [17]	2020	1	1 M	57		IIIb			
Li [18]	2020	28	28 M 20 F *	63 *^#^	23.7 *^#^	IIIa 11 IIIb 17			
Cillo [19]	2021	4	2 M 2 F	60 ^#^		IIIb 4	1	1	0/4
Sucandy [20]	2021	1	1 F	77		IIIb	0	1	
Di Benedetto [21]	2022	1	1 F	74		IIIa	0		1 ALPSS
Camerlo [22]	2022	1	1 F	56		IIIb	0	0	0/1
Deng [23]	2022	1	1 M	32		IV	0	0	

Abbreviations: BMI, Body Mass Index; BD, biliary drain; PVE, portal vein embolization; ALPSS, Associating Liver Partition and portal vein ligation for staged hepatectomy; * whole series; ^#^ mean value.

**Table 2:** Studies on robotic surgery for III–IV HC: study, patients and procedural characteristics.

Author	No. of Robtrocar	da Vinci Version	Type of Surgery	No. of Biliaryanastomosis	Operative Time (min)	Blood Loss (mL)	Conversion Rate	Length of Hospital Stay (Days)	Complications
Giulianotti [12]	4		RH + S1 + BR	1	540	800	0/1	11	0/1
Zhu [13]			RH + S1 + BR			700	0/1	14	0/1
Xu [14]	4	S	RH + S1 + BR 5 LH + S1 + BR 4		701 ^#^	1350 ^#^	0/4	20 ^#^	8/9
Quijano [15]	4	Si	LH + S1 + BR		510	1000	1/1	16	1/1
Machado [16]	4	Si	LH + S1 + BR	1	480	750	0/1		1/1
Marino [17]	4	Xi	LH + S1 + BR	1	295	280	0/1	6	0/1
Li [18]	4	Si	RH/LH + S1 + BR		276 *^$^	150 *^#^	0/28	9 *^$^	28/48 *
Cillo [19]	4	Xi	LH + S1 + BR 4	2.5 ^#^	850 ^#^	700 ^#^	1/4	9 ^#^	3/4
Sucandy [20]		Xi	LH + S1 + BR	1			0/1	6	
Di Benedetto [21]	4	Si	RH + S1 + BR	2	370	450	0/1	19	0/1
Camerlo [22]	4	X	LH + S1 + BR	2	420	100	0/1	8	0/1
Deng [23]	4		TMH + S + BR	2	340	100	0/1	17	0/1

Abbreviations: RH, Right Hepatectomy; LH, Left Hepatectomy; TMH, Taj Mahal Hepatectomy; BR, Biliary Reconstruction; * whole series; ^#^ mean value; ^$^ median value.

**Table 3:** Studies on robotic surgery for III–IV HC: study, patients and procedural characteristics.

Author	30-Day Mortality	Re-Operation	No. of Lymphnodes	R0 Resection	Pathologicalstaging	Follow-Up	Recurrance
Giulianotti [12]	0/1	0/1		1/1	T2N0	8	0/1
Zhu [13]	0/1	0/1		1/1			
Xu [14]	1/9	2/9		6/9		21.4 ^#^	8/9
Quijano [15]	0/1	0/1	13	1/1	T2N0		
Machado [16]	0/1	0/1		1/1	T1N0	5	0/1
Marino [17]	0/1	0/1	9	1/1			
Li [18]	0/28	0/28		35/48 *			
Cillo [19]	0/4	0/1	10 ^#^	3/4		8 ^#^	1/4
Sucandy [20]	0/1	0/1		1/1		12	0/1
Di Benedetto [21]	0/1	0/1	21	0/1	T4N1	13	0/1
Camerlo [22]	0/1	0/1	8	1/1	T2N1, T3Nx, T4N0, T4N1		
Deng [23]	0/1	0/1		1/1	T3N2	3	0/1

Abbreviations: * whole series; ^#^ mean value.

## Data Availability

Data are contained within the article.
